# Efficient generation of double heterologous promoter controlled oncolytic adenovirus vectors by a single homologous recombination step in Escherichia coli

**DOI:** 10.1186/1472-6750-6-36

**Published:** 2006-08-03

**Authors:** Dennis Hoffmann, Oliver Wildner

**Affiliations:** 1 Ruhr-University Bochum, Institute of Microbiology and Hygiene, Department of Molecular and Medical Virology, Bldg. MA, Rm. 6/40, D-44801 Bochum, Germany

## Abstract

**Background:**

Oncolytic adenoviruses are promising agents for the multimodal treatment of cancer. However, tumor-selectivity is crucial for their applicability in patients. Recent studies by several groups demonstrated that oncolytic adenoviruses with tumor-/tissue-specific expression of the E1 and E4 genes, which are pivotal for adenoviral replication, have a specificity profile that is superior to viruses that solely target the expression of E1 or E4 genes. Presently the E1 and E4 regions are modified in a time consuming sequential fashion.

**Results:**

Based on the widely used adenoviral cloning system AdEasy we generated a novel transfer vector that allows efficient and rapid generation of conditionally replication-competent adenovirus type 5 based vectors with the viral E1 and E4 genes under the transcriptional control of heterologous promoters. For insertion of the promoters of interest our transfer vector has two unique multiple cloning sites. Additionally, our shuttle plasmid allows encoding of a transgene within the E1A transcription unit. The modifications, including E1 mutations, are introduced into the adenoviral genome by a single homologous recombination step in *Escherichia coli*. Subsequently infectious viruses are rescued from plasmids. As a proof-of-concept we generated two conditionally replication-competent adenoviruses Ad.Ki•COX and Ad.COX•Ki with the promoters of the Ki-67 protein and the cyclooxygenase-2 (COX-2) driving E1 and E4 and vice versa.

**Conclusion:**

We demonstrated with our cloning system efficient generation of double heterologous promoter controlled oncolytic adenoviral vectors by a single homologous recombination step in bacteria. The generated viruses showed preferential replication in tumor cells and in a subcutaneous HT-29 colon cancer xenograft model the viruses demonstrated significant oncolytic activity comparable with *dl*327.

## Background

Human adenoviruses normally infect a wide variety of both dividing and nondividing cells. They replicate in and lyse their host cells to release newly generated progeny virus. Although these properties are ideal for killing human cancer cells, virus replication must be restricted to malignant cells preferably without toxicity to normal surrounding cells. The clinical relevance is emphasized by the lack of an established effective therapy for serious, disseminated adenovirus infection [1,2]. Oncolytic adenoviruses can be armed by therapeutic transgenes to augment their anticancer capacity [3-5]. Furthermore they can enhance synergistically chemotherapy and/or radiation in a multimodal anti-neoplastic approach [3,6,7]. To improve the safety and the therapeutic index of replication-competent adenoviral vectors, many unique strategies have been developed in an attempt to achieve tumor selectivity [8]. In one approach, viral genes that are essential for viral replication are placed under the control of promoters that are preferentially active in tumor cells compared to the surrounding normal tissues.

After adenoviral infection, E1A is the first viral gene expressed, and its product trans-activates the other promoters of early genes allowing expression of late genes, upon which viral replication depends [9]. Thus, the most commonly used strategy to construct conditionally replication-competent adenoviruses is to place only the adenoviral E1A region under the control of tissue/tumor-specific promoters [10-13]. Since already small amounts of E1A gene products are sufficient to initiate adenoviral replication resulting in the accumulation of E1A gene products and thus driving viral replication [14], leaky replication is frequently observed with this strategy. Controlling both E1A and E1B genes with a tissue/tumor-specific promoter improves the tissue/tumor-specific replication of the virus [15].

Like the E1 gene products, the adenoviral E4 gene region is expressed early after infection and is an essential component of the viral life cycle. The adenoviral E1 and E4 gene products function in concert to create a cellular environment permissive for efficient expression and processing of viral gene products and ultimately a productive virus infection [16].

Several groups reported that controlling simultaneously the expression of E1A and E4 genes will provide a tighter control over virus replication [17-22] and at the same time decreases the risk of generation of wild-type revertants. Because of the difficulty in finding two active and tightly regulated promoters for a certain tumor type that maintain their specificity in the adenoviral genome [23] several groups placed the E1 and E4 genes under the control of the same promoter [19-21]. Others used two different heterologous promoters to control E1 and E4 expression [17,18,22].

In a previous study [22] we demonstrated that transcriptional targeting of solitary E1A resulted in ~50% improved vector targeting when compared to an unrestricted replication-competent adenovirus. Furthermore, we showed that transcriptional targeting of adenoviral E1A and E4 with different promoters enhances vector targeting when compared to vectors using the same promoter.

In these studies, E1 and E4 double heterologous promoter controlled adenoviruses were generated by a lengthy sequential multistep cloning procedure to replace the viral E1 and E4 promoter [19,20,22]. Another approach in E. *coli *involves a multistep modification of the transfer vector which had to be done for each inserted heterologous promoter individually [18]. Aside from recombination systems in E. *coli *[24,25] the adenoviral genome can also be modified efficiently in yeast [26,27]. Fuerer *et al. *used a multistep recombination system in yeast to introduce multiple heterologous promoters/enhancers in the adenoviral genome [17].

Since among current methods there is no simple procedure to generate replication-competent adenoviral vectors with double heterologous promoters driving the viral E1 and E4 region, we developed based on the widely used adenoviral cloning system AdEasy [24,25] a transfer vector with two unique multiple cloning allowing the convenient insertion of heterologous promoters to transcriptionally target the expression of adenoviral E1 and E4 genes. In addition, the new transfer vector can accommodate a transgene, which is followed by an internal ribosomal entry site (IRES) upstream of the viral E1A transcription unit. This design has been used by us previously to generate replication competent adenoviral vectors encoding the herpes simplex virus thymidine kinase (HSV-TK), green fluorescent protein (GFP) or luciferase [2,4,28,29]. In the meantime we generated also double heterologous promoter controlled conditionally replication-competent adenoviral vectors encompassing a 24 bp deletion in the E1A conserved region 2 domain (E1Δ24), corresponding to amino acids L_122_TCHEAGF_129_, that is required for retinoblastoma protein (pRB) binding and release of E2F from pRb, which is associated with cell proliferation [30].

To evaluate the utility of our technology, we replaced the adenoviral E1 and E4 promoter with the COX-2 or Ki-67 promoter, respectively. Cyclooxygenase-2 (COX-2), which is primarily responsible for prostaglandins produced in inflammatory sites and is virtually undetectable in most tissues under physiological conditions, is upregulated in breast, bladder, lung, pancreas, as well as colon cancer [31]. The Ki-67 protein is exclusively expressed in proliferating cells and is therefore an important diagnostic tool for the detection of malignancies [32]. Previously we demonstrated that the COX-2 and Ki-67 promoter maintains their specificity in the adenoviral context [23]. In contrast to the often used cell cycle dependent E2F1 promoter, the activity of the Ki-67 promoter is not influenced by the adenoviral E1A gene products, which release free E2F by interacting with pRB [33].

In this proof-of-concept study we demonstrated efficient and straightforward generation of oncolytic adenoviruses with E1 and E4 driven by heterologous promoters. We evaluated the adenoviruses Ad.Ki•COX and Ad.COX•Ki *in vitro *and *in vivo*. We will make the shuttle plasmids available to the scientific community for noncommercial purposes.

## Results

**Figure 1:**
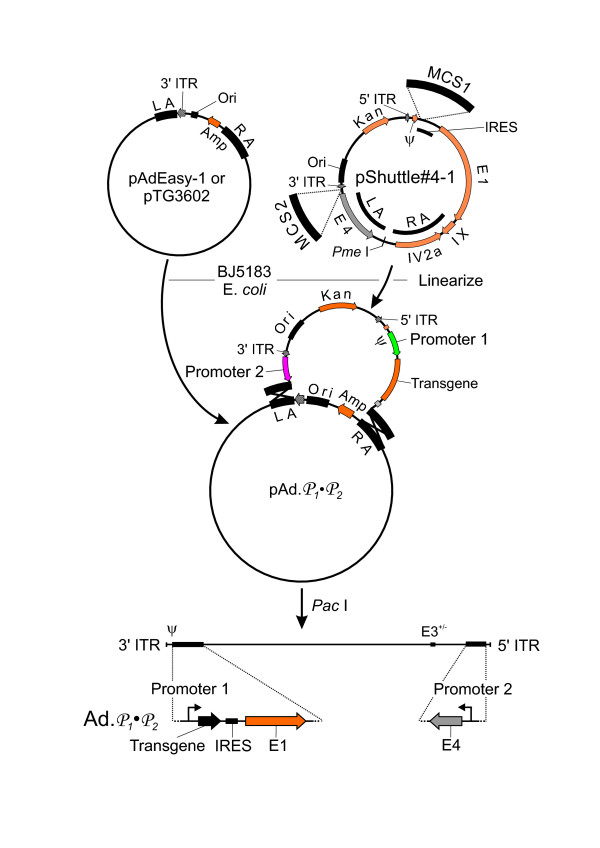
**Strategy outline**. To generate E1 and E4 heterologous promoter controlled replication-competent adenoviral vectors, the promoters of interest are inserted into the appropriate multiple cloning sites (MCS) of the transfer vector pShuttle#4-1 (or pShuttle#4-1Δ24). E. *coli *BJ5183 cells are transformed with the adenoviral backbone plasmid pAdEasy-1 or pTG3602 and the linearized transfer vector. Recombinants resistant to kanamycin are analyzed by restriction enzyme analysis to determine whether homologous recombination occurred at the corresponding "left arm" and "right arm". Subsequently infectious recombinant adenovirus is rescued from 293 cells.

We constructed two shuttle plasmids with wild-type adenovirus E1 and the mutant E1Δ24 [33] both allowing in a single homologous recombination step in E. *coli *to replace the viral E1 and E4 promoter with heterologous promoters (strategy outlined in Fig. 1). In this proof-of-concept study we evaluated the transfer vector pShuttle#4-1 with wild-type E1 by generating two replication-competent adenoviruses Ad.Ki•COX and Ad.COX•Ki based on the pAdEasy-1 adenoviral backbone.

### Analysis of promoter activity

**Figure 2:**
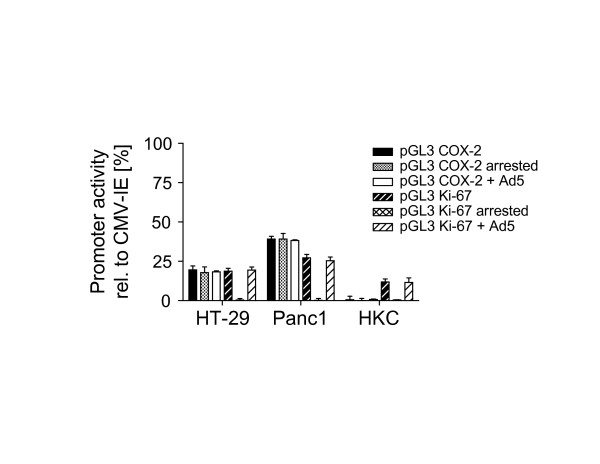
**Luciferase reporter assays**. The specificity of the COX-2 and Ki-67 promoter was confirmed using a dual luciferase reporter assay, allowing the analysis of test promoters *via firefly *luciferase activity and normalization of transfection efficiency *via Renilla reniformis *luciferase driven by the constitutive CMV-IE promoter in a single-well. Promoter activity was measured in subconfluent or G0/G1-arrested cells. To examine whether adenoviral gene products influence the promoter activity cells were infected with Ad5 after transfection with the reporter plasmids. The activity of the COX-2 and Ki-67 promoter was normalized to that of the CMV-IE promoter. Data are presented as mean ± SD from four independent experiments.

Before inserting the promoters for the COX-2 and Ki-67 gene into our transfer vector pShuttle#4-1, we confirmed the specificity of these promoters. For this we conducted transient dual luciferase reporter gene assays in the colorectal cancer cell line HT-29 and the pancreatic cancer cell line Panc1. Primary normal human keratinocytes (HKC) served as a control. As shown in Fig. 2, when compared to the CMV-IE promoter, the median activity of the COX-2 promoter in these cells (subconfluent) was 19.5%, 39.1%, and 0.5%, respectively. In cells arrested in G0/G1 or infected with Ad5, the activity of the COX-2 promoter did not change significantly (*P *= NS, Student's t test). When compared to the CMV-IE promoter, the median activity of the Ki-67 promoter in subconfluent cells was 18.7%, 27.2%, and 11.9%, respectively. Infection of the cells with Ad5 had no significant effects on the activity of the Ki-67 promoter (*P *= NS, Student's t test). However, the activity of the Ki-67 promoter was reduced to background levels in G0/G1 arrested cells (*P *< 0.001, Student's t test). Flow cytometric analysis of cell cycle distribution revealed that ~96% of the cells were in G0/G1 (data not shown).

### Efficient generation of double heterologous promoter controlled adenoviruses

To place the adenoviral E1 and E4 genes under the transcriptional control of the COX-2 and Ki-67 promoter we inserted these promoters in the appropriate multiple cloning site of our transfer vector generating pShuttle#4 COX•Ki and pShuttle#4 Ki•COX. To reduce promoter interference, we placed upstream of the promoters a synthetic polyA site [23].

**Figure 3:**
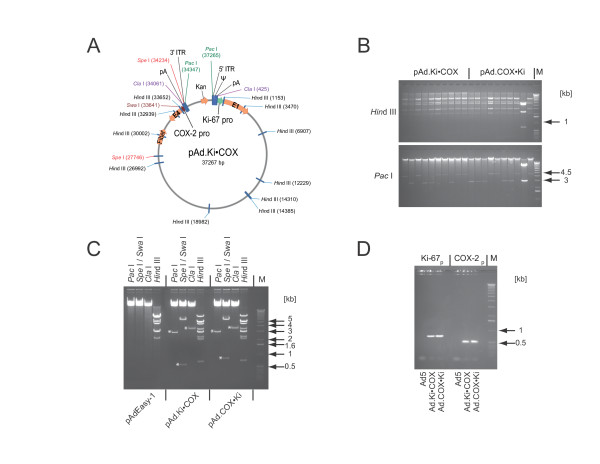
**Cloning of oncolytic adenoviruses Ad.Ki•COX and Ad.COX•Ki**. **Panel A: **The structure of the recombined adenoviral plasmid pAd.Ki•COX is shown. Restriction endonucleases used for analysis are mapped. For clarity, a map of pAd.COX•Ki, which is with the exception of the swapped promoters identical to pAd.Ki•COX, has been omitted. **Panel B: **BJ5183 cells pre-transformed with pAdEasy-1 were transformed with pShuttle#4 Ki•COX and pShuttle#4 COX•Ki. The integrity of the pAdEasy-1 plasmid in the pre-transformed E. *coli *was confirmed by *Hin*d III digestion (data not shown). For each construct ten small scale DNA plasmid preparations of kanamycin resistant clones were digested with *Hin*d III and *Pac *I and analyzed by electrophoresis through a 0.8% agarose gel and ethidium bromide staining. **Panel C: **High quality DNA was prepared by CsCl banding of potential valid recombinants and digested with *Pac *I, *Spe *I/*Swa *I, *Cla *I, and *Hin*d III. The heterologous promoter driving the viral E4 region was flanked by *Spe *I and *Swa *I and the promoter controlling E1 by *Cla *I. The adenoviral backbone plasmid pAdEasy-1 served as a control. Diagnostic fragments obtained with each enzyme are marked with an asterisk (*). **Panel D: **Standard PCR was used to confirm on the rescued infectious recombinant adenoviruses Ad.Ki•COX and Ad.COX•Ki the presence of the Ki-67 and COX-2 promoter with the upstream polyA sequences in each virus. Ad5 served as negative control. The 1 kb DNA ladder from Invitrogen/Gibco was used as DNA size marker (M).

After linearization of the transfer vectors, they were recombined with the adenoviral backbone plasmid pAdEasy-1 *via *their homologous DNA in E. coli. The structure of the recombined plasmid pAd.Ki•COX with cleavage sites for the restriction endonucleases used for analysis is shown in Fig. 3A. The plasmid pAd.Cox•Ki is identical with the exception of the promoters for E1 and E4. As shown in Fig. 3B, restriction analysis of kanamycin resistant clones with *Hin*d III demonstrated that only one out of 20 analyzed clones resulted from non-recombined transfer vector. Restriction of the adenoviral plasmids pAd.Ki•COX and pAd.COX•Ki with *Pac *I revealed that in 4 out of 10 and in 5 out of 9 recombinants homologous recombination occurred between the corresponding arms of shuttle and adenoviral backbone plasmid. In the other cases, recombination occurred between the *ori *sequences shared by the transfer vector and the adenoviral backbone plasmid pAdEasy-1 and the corresponding right arms. In these clones the native viral E4 promoter has not been replaced with the heterologous promoter of interest.

As shown in Fig. 3C we further analyzed the recombinants pAd.Ki•COX and pAd.COX•Ki by *Spe *I and *Swa *I double digestion to release the heterologous promoter driving the viral E4 region. Electrophoretic analysis resulted in a fragment in the length of the COX-2 promoter (597 bp) and Ki-67 promoter (814 bp) with upstream polyA, as well as in a 31.1 kb and a 5.9 kb fragment. The heterologous promoter driving the viral E1 region is incorporated in the ~3.5 kb *Cla *I fragment. Because neither pAdEasy-1 nor the shuttle plasmid contains the native viral E1 promoter, recombinants encoding E1 under the transcriptional control of the native viral E1 promoters are not possible in *E. coli*.

Since both the COX-2 and the Ki-67 promoter were active in subconfluent 293 cells, we used these cells to rescue recombinant replication-competent adenoviruses, as described previously [25]. As shown in Fig. 3D we verified the genomic integrity of the rescued infectious adenoviruses Ad.Ki• COX and Ad.COX•Ki by PCR amplification of the Ki-67 and COX-2 promoter sequences with upstream polyA. The size of the PCR amplified fragments were 792 bp and 582 bp, respectively, as expected. Ad5 served as negative control.

### Adenoviral cytopathic effect assay

We determined the Ad5 transduction efficiency of all cell lines used in this study. For this cell monolayers were incubated for 24 hours with Ad.GFP at an MOI of 0.5 PFU/cell (determined on 293 cells by TCID_50_). In HT-29, Panc1, PAC and 293 cells the median fraction of GFP positive was ~45%. Under the same conditions, the median transduction efficiency of PPC and primary human keratinocytes (HKC) was 21.6% and 9.3%, respectively.

Since all vectors are based on Ad5 and thus penton base and fiber which determine the infection efficiency on the viral side are identical, we used the transduction efficiency with Ad.GFP for individual infection conditions for each cell line to compensate for differences in adenoviral transduction efficiency. With the Ad.GFP vector we verified the applicability of our predicted compensation (data not shown).

**Figure 4:**
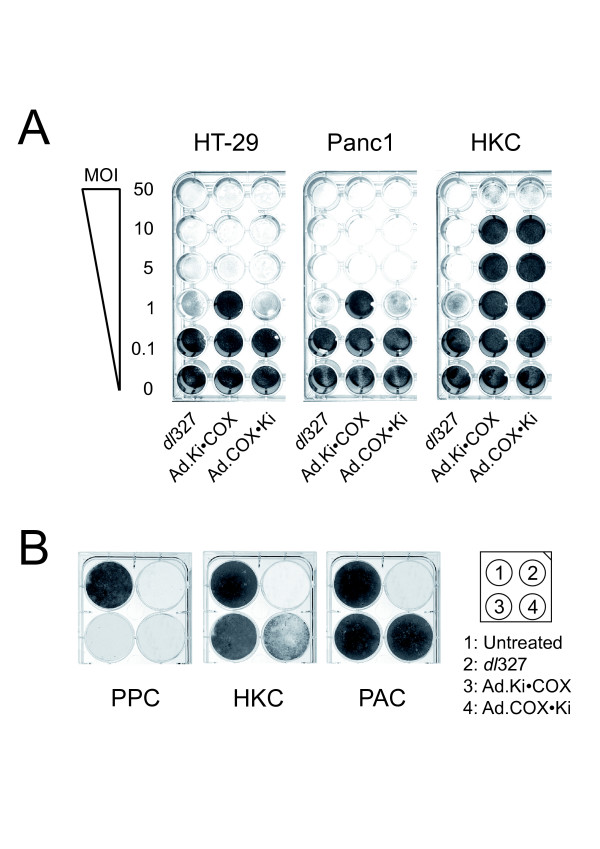
**Adenoviral cytopathic effect assay**. **Panel A: **Monolayers of HT-29, Panc1, and primary human keratinocytes (HKC) were infected with serial dilutions of *dl*327, and adenoviral vectors carrying the adenoviral E1A region and E4 under the transcriptional control of the COX-2 or Ki-67 promoter, respectively. The indicated MOI (PFU/cell) was determined for each cell line individually. **Panel B: **monolayers of primary pancreatic carcinoma cells (PPC), HKC, and primary adenoid cells (PAC) were left untreated or infected at an MOI of 5 PFU/cell (determined for each cell line individually) with *dl*327, Ad.Ki•COX or Ad.COX•Ki, respectively. Five days later, the cells were washed and stained. One representative experiment out of three is shown.

To determine the adenoviral cytopathic effect, monolayers of HT-29, Panc1 and HKC were infected with serial dilutions of *dl*327, Ad.Ki•COX and Ad.COX•Ki. As shown in Fig. 4A, Ad.Ki•COX and Ad.COX•Ki demonstrated lysis of HT-29 and Panc1 cell monolayers. The lytic effect of Ad.COX•Ki in both cell lines tested was slightly greater than that of Ad.Ki•COX. Furthermore, the lytic effect of the recombinant adenoviruses in HT-29 and Panc1 was similar to that of *dl*327. In contrast, in primary human keratinocytes, the cytopathic effect of the adenoviruses Ad.Ki•COX and Ad.COX•Ki was greatly reduced when compared to *dl*327.

Since permanent cell lines might have changed their characteristics over time, we verified our cytopathic effect assay also in primary pancreatic carcinoma cells (PPC). As a control for normal cells we used primary adenoid cells (PAC) in addition to HKC (Fig. 4B). Infection of PPC cells with *dl*327, Ad.Ki•COX, or Ad.COX•Ki resulted in complete lysis of the cell monolayers. Furthermore, infection of the non-neoplastic HKC or PAC cells with *dl*327 caused complete cytopathic effect. In contrast, infection of the monolayers with the viruses Ad.Ki•COX or Ad.COX•Ki did not cause any significant cell lysis, with the exception of Ad.COX•Ki in HKC cells where a slight cytopathic effect was visible.

### *In vivo *evaluation of the oncolytic adenoviruses

**Figure 5:**
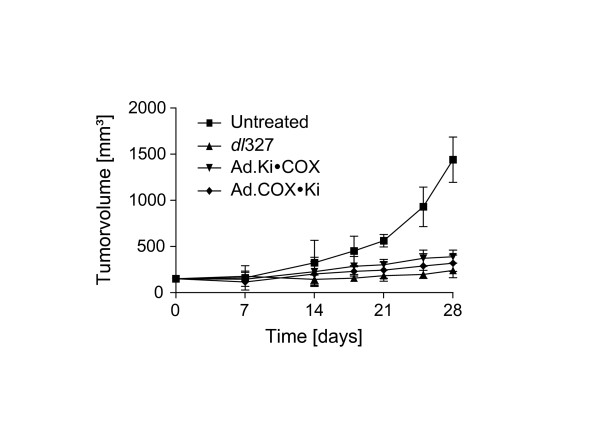
**Anti-neoplastic efficacy of adenoviruses**. HT-29 xenografts were grown subcutaneously to volumes of ~200 mm^3^. Groups of mice (n = 8) were treated with indicated adenoviruses by intratumoral injection of 5 × 10^9 ^virus particles in 100 μl PBS on day 0 and day 2. Tumors were measured at indicated time points and calculated volumes (mean ± SD) are presented as growth curves.

Furthermore we evaluated in an HT-29 subcutaneous colon carcinoma model in nude mice the oncolytic efficacy of the double heterologous promoter controlled vectors (Ad.Ki•COX, and Ad.COX•Ki) and the adenoviral E3-deletion mutant *dl*327. As shown in Fig. 5, on day 28, the median tumor volume of untreated animals was 1408.6 mm^3^. The median tumor volume of animals treated with *dl*327, Ad.Ki•COX, or Ad.COX•Ki was 241.7 mm^3^, 383.8 mm^3^, and 290.7 mm^3^, respectively.

One-Way Analysis of Variance (ANOVA) with Tukey's HSD post hoc test revealed that the tumor volumes of all treatment groups receiving adenovirus were significantly smaller when compared to untreated animals (*P *< 0.0001). Furthermore there was no statistical significant difference in the treatment efficacy of Ad.Ki•COX, Ad.COX•Ki and *dl*327 (*P *= NS).

## Discussion

In the context of conditionally replication-competent adenoviral vectors, several groups reported that dual transcriptional targeting of viral E1A and E4 genes allows a significantly higher level of tumor specificity than solely targeting E1A or E4 genes [17-22]. In these studies, E1 and E4 double heterologous promoter controlled adenoviruses were generated by a timely multistep cloning procedure to replace the viral E1A and E4 promoter by tumor-/tissue-specific promoters in a sequential fashion. In this proof-of-concept study we demonstrated efficient and straightforward generation of conditionally replication-competent adenoviruses with E1 and E4 genes driven by heterologous promoters.

Previous studies demonstrated that not all promoters are suitable for the transcriptional targeting of adenoviral E4 gene products [20,34] and that heterologous promoters might lose their specificity in the adenoviral context [35,36]. Therefore it is likely that several adenoviral vectors with different heterologous promoters have to be generated and evaluated for their tumor specificity. Thus our system will be valuable for the generation of conditionally replication-competent adenoviral vectors with E1 and E4 driven by tumor-/tissue-specific promoters.

Dependent on the used adenoviral backbone plasmid, *i.e*. pAdEasy-1 [25] or pTG3602 [24], our system allows generation of E3-deleted or wild-type E3 vectors.

Deletion of the adenoviral E3 region allows insertion of larger promoters and/or transgenes but reduces also the intrinsic oncolytic capacity of these vectors [37,38].

Analogous to our oncolytic adenovirus vectors encoding HSV-TK, GFP or luciferase [2,4,28,29], our system allows the incorporation of a transgene into the E1A transcription unit. This design allows high transgene expression. Since the level of E1A expression has only a slight effect on virus replication [14], in these vectors the replication was not significantly affected. In addition, mutations of the E1 region, *e.g*. ΔE1B-55K [39] or E1Δ24/*dl*922-947 [33], can be easily incorporated into this system as long as this region is not present in the used adenoviral backbone plasmid (*e.g*. Ad.Easy-1).

As a proof-of-concept we generated with our novel transfer vector two adenoviruses Ad.Ki•COX and Ad.COX•Ki with the promoters of the Ki-67 protein and the cyclooxygenase-2 (COX-2) driving E1 and E4 and vice versa. We choose the COX-2 and Ki-67 promoter since we did know from previous studies that these promoters maintain their specificity in the adenoviral genome and in the presence of adenoviral gene products [23]. Furthermore, since for most tumors it is difficult to specify a truly tumor specific promoter we combined the tumor-/tissue-specific promoter COX-2 with a proliferation-associated Ki-67 promoter to target viral replication and lysis to the transcriptional intersection of these two promoters.

The COX-2 promoter was active in the tested colon and pancreatic cancer cell lines, but not in human keratinocytes which we used as a control for normal cells. In contrast to the E2F promoter often used in oncolytic adenoviral vectors [11,19], the Ki-67 promoter is not activated in plasmid based reporter gene assays when over infected with wild-type Ad5 [23]. To our knowledge this is the first report using this promoter in oncolytic adenoviral vectors.

In ~50% of the recombinants generated with our novel shuttle vector, the viral E4 promoter was replaced with the heterologous E4 promoter. In recombinants generated by homologous recombination of the shuttle plasmid and pAdEasy-1 *ori *sequences the viral E4 promoter will not be substituted with the heterologous promoter. The efficiency was influenced by the promoter sequence and length. Up to now we were able to replace the viral E4 promoter efficiently with heterologous promoters of up to 1.5 kb in length. Using the adenoviral backbone plasmid pAdEasy-1 or pTG3602, heterologous sequences with a total length of up to 4.4 kb or 2.6 kb, respectively, can be inserted [40].

*In vitro*, the constructed double heterologous promoter controlled replication-competent adenoviral vectors Ad.Ki•COX and Ad.COX•Ki demonstrated efficient lysis of the tested colon and pancreas cancer cell lines, but poor lysis in primary human keratinocytes (HKC). In contrast, the cytopathic effect caused by *dl327 *infection was similar in all cell lines tested. Since permanent cell lines have been often in continuous culture for several years and thus are likely to have genetic aberrations, we established short-term cultures of primary pancreatic cancer cells. Both recombinant adenoviruses and *dl327 *demonstrated efficient cell lysis of these cells. In contrast, Ad.Ki•COX and Ad.COX•Ki showed greatly reduced cell lysis in short-term cultures of primary adenoid cells and keratinocytes when compared to *dl327*. Because we compared the cytopathic effect of the Ad.Ki•COX and Ad.COX•Ki vectors to *dl327*, we put special emphasis on the titer determination of *dl327*, since an underestimation of *dl327 *titer would have put the specificity of the vectors Ad.Ki•COX and Ad.COX•Ki into doubt.

Furthermore, since in all adenoviruses the E3-11.6K protein (ADP) was deleted, which is required for efficient cells lysis and release of adenoviral progeny from infected cells [37] and thus resulting in reduced viral replication kinetics when compared to wild-type Ad5 [28], the differences in cytopathic effect seen are clearly due to the promoter substitution.

We also evaluated in a subcutaneous HT-29 colon xenograft model in nude mice the oncolytic activity of the viruses Ad.Ki•COX, Ad.COX•Ki and *dl327*. The key findings were: First, intratumoral injections of replication-competent adenoviruses resulted in a significant anti-tumor response when compared to untreated animals, confirming that replication-competent adenoviruses have a direct oncolytic activity [39,41]. Second, the anti-neoplastic efficacy of the unrestricted replication-competent *dl*327 virus was similar to that of the vectors Ad.Ki•COX and Ad.COX•Ki. Third, although at any given time only a fraction of tumor cells are in S phase there was no significant difference in the anti-neoplastic efficacy of Ad.Ki•COX and Ad.COX•Ki.

## Conclusion

We demonstrated in our proof-of-concept study that our novel transfer vector allows efficient and straight forward generation of double heterologous promoter controlled adenoviruses in a single homologous recombination step in bacteria. Dependent on the used adenoviral backbone plasmid, *i.e*. pAdEasy-1 or pTG3602, our system allows generation of E3-deleted or wild-type E3 vectors. The rescued infectious adenoviruses demonstrated preferential lysis of tumor cells, thus confirming previous reports on restriction of adenoviral replication by transcriptional targeting of the viral E1A and E4 region. The use of tumor-specific promoters to control the expression of viral genes pivotal for replication is a promising method for the construction of therapeutic viruses.

## Methods

### Cells and cell culture

The cell lines HT-29 (HTB-38), Panc1 (CRL-1469), and 293 (CRL 1573) were obtained from the American Type Culture Collection (Manassas, VA) and propagated in D-10 medium, consisting of high glucose DMEM medium supplemented with 10% heat-inactivated fetal bovine serum, 50 μg/ml gentamicin, and 2 mM glutamine (Invitrogen/Gibco, Paisley UK). Primary human keratinocytes (HKC) were kindly provided by K. Reimers (Klinik für Plastische, Hand- und Wiederherstellungschirurgie, MHH, Hannover, Germany) and propagated in PromoCell Keratinocyte Growth Medium 2 (Promocell GmbH, Heidelberg, Germany). As an additional control for normal cells, we established short term cultures of adenoid cells, designated PAC, from children who underwent tonsillectomy for medical reasons. Since permanent tumor cell lines might have changed their characteristics during the course of continuous culture, we established also short-term cultures of pancreatic cancer cells, designated PPC, from patients treated surgically for histologically confirmed pancreatic cancer. Ethical clearance was obtained from the local Ethics Committee (Reg. No. 2487). Cells were obtained from tissue by mechanical disruption and enzymatic digestion with DNAse I (80 mg/ml), hyaluronidase (2000 U/ml) and collagenase (40 mg/ml) (Sigma-Aldrich, St. Louis, MO). Cells were grown in DMEM/F12 (1:1) medium supplemented with 10% heat-inactivated fetal bovine serum, 50 μg/ml gentamicin, and 2 mM glutamine. All cell lines were maintained at 37°C in a humidified atmosphere of 95% air and 5% CO_2_.

### Plasmids, DNA transfections, and luciferase reporter assays

The COX-2 (-327/+59) promoter fragment [42] was PCR amplified with the primers 5'-GAGCTCGAAGAAGAAAAGACATCTGGCG-3' and 5'-CTCGAGGCGCTGCTGAGGAGTTCCTG-3' and subsequently inserted into the *Sac *I/*Xho *I sites (underlined in the primer sequence) of the luciferase reporter vector pGL3-Basic (Promega, Madison, WI). The Ki-67 (-600/+7) promoter fragment [43] was PCR amplified using the primers 5'-CTCGAGGATAGCAGGACGGGTTATCT-3' and 5'-AAGCTTTTCCACTTGTCGAACCACCG-3' and cloned into the *Xho *I/*Hin*d III sites (underlined in the primer sequence) of pGL3-Basic. Promoter studies were carried out using a dual luciferase reporter assay (Promega), allowing the analysis of test promoters *via firefly *luciferase activity and normalization of transfection efficiency *via Renilla reniformis *luciferase driven by the constitutive CMV-IE promoter in a single-well. For promoter analysis in subconfluent cell monolayers, cells were seeded one day prior to transfection at a density of 1 × 10^4 ^cells per well into 24-well tissue culture plates. Cell monolayers were co-transfected with 9.9 μg *firefly *reporter vector DNA and 0.1 μg DNA of the *Renilla *luciferase expression plasmid pRL-CMV (Promega) using the calcium phosphate method [44].

To determine whether adenovirus early gene products influence the activity of the tested promoters, cells were infected with *dl327 *at a multiplicity of infection (MOI) of 5 PFU/cell 36 hours after transfection with reporter gene plasmids. Forty-eight hours after transfection, cell lysates were analyzed using a microplate luminometer (Berthold ORION-96, Bad Wildbad, Germany), as described previously [23]. Studies were carried out in four independent experiments using two preparations of plasmid DNA from the *E. coli *strains DH5α and Stbl2 (Invitrogen/Gibco).

### Arrest and analysis of cell cycle

Forty-two hours after transfection cells were arrested in G0/G1 by UV irradiation (25 J/m^2^) using a Stratalinker 1800 UV Crosslinker (Stratagene, La Jolla, CA), as described previously [45]. The dose of UV radiation used was optimized for best cell cycle arrest with minimal induction of apoptosis. Six hours later, luciferase reporter gene assays and flow cytometric cell cycle analysis were carried out as described previously [23].

### Adenoviral cloning system

Our cloning system consists of two plasmid vectors: The pAdEasy-1 adenoviral backbone plasmid, provided by Bert Vogelstein (Johns Hopkins Kimmel Cancer Center, Baltimore, MD) [25] contains the entire adenovirus type 5 (Ad5) genome except nucleotides 1 – 3,533 (encompassing the E1 region) and nucleotides 28,130 – 30,820 (encoding E3).

To increase the probability of generating recombinant plasmids containing the recombinant adenoviral genome, we used BJ5183 cells carrying intact pAdEasy-1 plasmid [46]. Since the large backbone is susceptible to damage during the transformation procedure, the used batch of pAdEasy-1 transformed BJ5183 cells was tested by restriction enzyme analysis and the ability to rescue infectious adenovirus from recombinant plasmids.

The new component is our transfer vector pShuttle#4-1, which encodes, in contrast to pShuttle [25], the entire Ad5 E1 region (right arm, Ad5 nucleotides 499 – 5767; for comparison pShuttle Ad5 nt 3,534–5,790). The viral E1A promoter has been replaced with a multiple cloning site containing a *Bam*H I, *Srf *I, *Not *I, *Xho *I, and *Sal *I restriction site to allow insertion of a heterologous promoter or promoter and transgene which is then transcriptionally linked with the adenoviral E1 gene through an internal ribosome entry site (IRES) sequence. Furthermore, the viral E4 promoter has been replaced with a second MCS with the restriction sites *Spe *I, *Nsp *V, *Swa *I and *Nhe *I. The left arm encompasses Ad5 nucleotides 33639 – 35617 (for comparison pShuttle 34,931–35,935). Left and right arm allow homologous recombination with the adenoviral plasmid (pAdEasy-1). The adenoviral 3' ITR, multiple cloning sites and appropriate restriction sites for inserting PCR cloned adenoviral arms were created by a 229 bp synthetic linker with the sequence GGTACCGGATCCGCCCGGGCGCGGCCGCCTCGAGGTCGACAACTTAAGGATATCGTATACGTTTAAACGAATTCATCGATTCTAGAGCTAGCATTTAAATTTCGAAACTAGTCTACGTCACCCGCCCCGTTCCCACGCCCCGCGCCACGTCACAAACTCCACCCCCTCATTATCATATTGGCTTCAATCCAAAATAAGGTATATTATTGATGATGTTAATTAACATATG inserted into the *Kpn *I and *Nde *I restriction sites of pShuttle, flanking 5' the adenoviral encapsidation signal (ψ) and 3' pBR322 origin of replication and kanamycin resistance gene.

To facilitate the insertion of promoters in our transfer vector pShuttle#4-1 from the reporter plasmid pGL3-Basic (Promega), we generated the plasmid pBS-linker, which contains between the *Sac *I – *Kpn *I site of pBS SK+ (Stratagene, La Jolla, CA) a multiple cloning site with the endonuclease restriction sites *Spe *I, *Nsp *V, *Not *I, *Eco*R V, *Hin*d III, *Sal *I, and *Swa *I. The COX-2 and Ki-67 promoters were inserted with the upstream synthetic polyA from pGL3-Basic as *Not *I – *Hin*d III fragments into the plasmid pBS-linker and then into pShuttle#4-1 as *Not *I – *Sal *I fragments for driving E1A and as *Spe *I – *Swa *I fragments into the second MCS to control transcription of E4.

As outlined in Fig. 1, after insertion of both promoters and facultative a transgene into pShuttle#4-1, the transfer vector needs to be linearized with *Pme *I (alternatively *Eco*R I or *Bst*1107 I). Subsequently pAdEasy-1 pre-transformed [46] electrocompetent E. *coli *BJ5183 cells are transformed with the linearized transfer vector. Alternatively pTG3602 can be used as adenoviral backbone. Second, recombinants were selected with kanamycin and screened by *Hin*d III restriction endonuclease digestion. Since the native viral E4 promoter will only be replaced with the heterologous promoter if the homologous recombination occurred between left and right arms of pAdEasy-1 and transfer vector, recombinants with complex *Hin*d III restriction pattern were digested with *Pac *I. If the homologous recombination occurred between the left arms a ~3 kb and ~35 kb fragment will result. However, if the recombination occurred through the plasmid *ori *sequences shared between the transfer and pAdEasy-1 vector a 4.5 kb and ~35 kb fragment will occur. The resulting adenoviral recombinants are single heterologous promoter controlled replication-competent vectors with a native viral E4 promoter. In the last step, recombinant adenoviral constructs are digested with *Pac *I to liberate ITRs and transfected into a production cell line, *i.e*. 293 cells. If the heterologous E4 promoter does not work in this cell line, the viral E4 function needs to be trans-complemented.

### Adenoviruses

The adenoviruses Ad.COX•Ki and Ad.Ki•COX with the COX-2 and Ki-67 promoter driving E1 and E4 and vice versa were generated as proof-of-concept with our novel transfer vector. The genomic integrity of Ad.COX•Ki and Ad.Ki•COX was verified by PCR for the presence of the Ki-67 and COX-2 promoter with upstream polyA. Adenoviral DNA was isolated using the Genomed JETQUICK Tissue Spin Kit (GENOMED, Löhne, Germany) according to the manufacturer's instructions. For amplification of polyA-Ki-67 promoter the primer pair GGCCGCAATAAAATATCTTT and CAAGCTTTTCCACTTGTCGA was used. The polyA-COX-2 promoter was amplified with the primer pair GCGGCCGCAATAAAATATCT and GTCGACAAGCTTACTTAGAT.

Adenoviral infection susceptibility of the cell lines was determined with Ad.GFP, a previously described vector encoding green fluorescence protein (GFP) driven by the CMV-IE promoter [47]. The E3-deleted Ad5 mutant *dl*327 [48] was used as unrestricted replication-competent adenovirus control, and the reference strain VR-5 (ATCC, Manassas, VA) was used as wild-type Ad5. Since in subconfluent 293 cells both the COX-2 and Ki-67 promoter [49] were active, all adenoviruses were amplified in 293 cells and purified by two rounds of CsCl density centrifugation and subsequent dialysis [50]. Concentration and bioactivity of the adenoviruses were determined by measuring absorbency at 260 nm and 50% tissue culture infective doses (TCID_50_) using 293 cells [51].

### Adenoviral infectivity assay

Cells were seeded (1 × 10^6 ^cells per well) into 6-well plates and 12 hours later incubated for 4 hours with Ad.GFP at a multiplicity of infection (MOI) of 5, 1, and 0.5 PFU per cell (determined on 293 cells). Twenty-four hours later, GFP expression was analyzed by flow cytometry (FACSCalibur flow cytometer, Becton Dickinson Immunocytometry Systems, Mansfield, MA)

### Cytotoxicity assay

To determine viral cytopathic effect, 2 × 10^5 ^and 1 × 10^6 ^cells were seeded into 24-well and 6-well plates. Cell monolayers were infected under the same conditions with the adenoviruses at an MOI of 0.1 to 50 and 5 PFU/cell in 1 ml or 5 ml D-10 growth medium, respectively. The dose of infection was adapted based on the transduction efficiency with an Ad5-based adenoviral vector encoding GFP. The infectious titer of Ad.GFP was determined by TCID_50 _on 293 cells. Four hours later, cell monolayers were washed and incubated with fresh growth medium. Within a day when complete lysis of monolayers infected with *dl*327 occurred (~5 days post infection) remaining cells were washed, fixed with 2% paraformaldehyde, subsequently stained with 1% crystal violet and photographed.

### Animal study

This study was approved by the local Animal Care and Use Committee. The anti-neoplastic efficacy of the adenoviral vectors was determined in a subcutaneous HT-29 tumor xenograft model in nude mice. For this, 5 to 7 weeks-old BALB/c *nu/nu *mice (Janvier, Le Genest-St-Isle, France) were injected with 1 × 10^7 ^HT-29 cells in 100 μl subcutaneously into the hind flanks. At least once a week, minimum and maximum perpendicular tumor axes were measured using vernier calipers, and tumor volume was calculated using the simplified formula of a rotational ellipse (*l *× *w*^2 ^× 0.5).

When the tumors reached a volume of ~200 mm^3^, animals were randomly assigned to treatment groups and received two intratumoral injections of 5 × 10^9 ^virus particles diluted in a volume of 100 μl phosphate-buffered saline (PBS) on day 0 and day 2. The untreated group received PBS.

### Statistical analysis

The software SPSS 12 (SPSS Inc., Chicago, IL) was used for statistical analysis with the indicated test.

## Authors' contributions

DH conceived and designed the study, performed the experiments and drafted the manuscript. OW carried out some of the experiments, participated in critical evaluation, coordination and funding for the study, and drafted the manuscript
